# Turning over New Leaves

**Published:** 1891-05-02

**Authors:** 


### Turning Over New Leaves.

### The Medical Annual.*

The *Medical Annual* for 1891 has been recently published. So far as we have seen, it is better than its immediate predecessors, and a great deal better than those more remote. It goes without saying that such a yearly résumé if well done is of very great service to the busy practitioner. His weekly medical paper is of course a constant stimulus and guide. But a compact and convenient work of reference like the *Annual* which deals exclusively with the treatment of diseases, and which is constantly brought down to date, is invaluable. Most of the writers' names are sufficient guarantee of the earnestness and solidity of their work. Dr. Graeme Hammond, of New York, endorses Dr. Seguin's somewhat startling boldness in the treatment of Chorea. Our mainstay, he says, must still be arsenic; and as quickly as possible we must get up to fifteen drop doses three times a day. It may be necessary then to call a halt for a few days. But we should resume where we left off, namely, at fifteen drops, and from that dose advance to twenty-five, or even twenty-seven, with all possible promptitude. We have not ourselves found it necessary to give such heroic doses as these; though we do not doubt that Dr. Hammond and Dr. Seguin are speaking from sufficient experience, and are right in their conclusions. It is highly probable that a cautious boldness in the administration of arsenic to Choreic cases will markedly shorten their duration, and please both the doctor and the patient. It seems a small matter to call attention to grammatical defects in an important work on medicine; but doctors are so notoriously careless of literary excellence that one cannot help feeling a certain degree of regret. Dr. Hammond, for example, has the following:—"Eye-strain is sometimes an accessory or secondary cause of much importance, and every choreic person should be tested for defects, and, if found, should be corrected." The grammar of this is that if the "person" be found, "he" should be corrected. What Dr. Hammond means is, that if any "defects of vision are found "they" should be corrected. The book, as we have said, is well up to date, excellently done, and is much enhanced in value by some very instructive diagrams. We cordially recommend it to all practitioners and students.

* The Medical Annual: Bristol, John Wright and Co.; London, Simpkin and Marshall and Hamilton and Kent.

